# Malaria Susceptibility—It's Not All in the Genes

**DOI:** 10.1371/journal.pmed.0020411

**Published:** 2005-11-08

Malaria parasites invade human red blood cells, and it had already been recognized in the 1940s that diseases of red blood cells such as thalassemia and sickle-cell anemia, which are the most common group of genetic disorders in humans, are mainly found in populations exposed to malaria and their descendants. It seems that much of the genetic variation that affects the phenotype of red blood cells appears to have evolved due to natural selection by malaria. Susceptibility to malaria is also thought to be determined by genetic variation in the human immune system. We know less about the specific immune system genes involved, but this is an important area of research because researchers hope that understanding the molecular basis of natural immunity will speed up the development of an efficient malaria vaccine.

Rather than focusing on the identification of specific genes and gene variants, Margaret J. Mackinnon and colleagues are interested in the relative contributions of host genetics and other factors to the risk of malaria. To estimate the overall contribution of genetic factors to the difference in disease incidence between individuals within a population, one needs three types of data: (1) disease incidence for individuals over a certain period of time (to be able to determine an individual's risk), (2) information on genetic relatedness of the individuals in the population, and (3) a setup in which individuals with different levels of relatedness share the same environment and/or where related individuals live in different environments. (The third condition is essential to distinguish between genetic and environmental effects.) [Fig pmed-0020411-g001]

**Figure pmed-0020411-g001:**
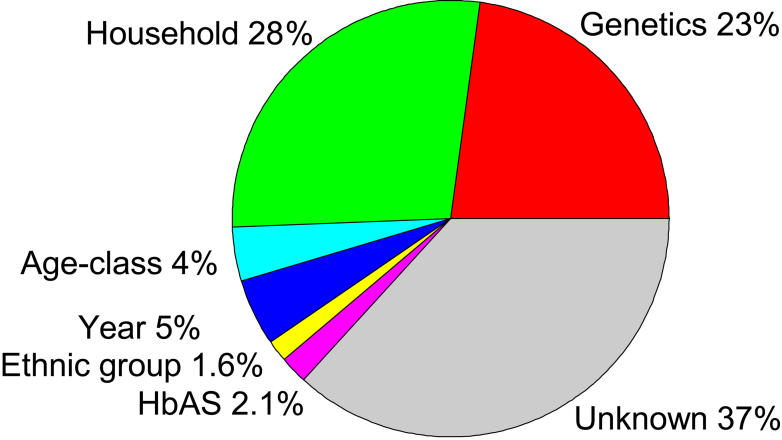
Contributors to variation in mild malaria risk

Mackinnon and colleagues studied two populations of children from a malaria-endemic area in Kenya for which they could obtain the necessary data. In one case, they determined incidence of mild clinical malaria in 640 children over a period of five years. Genetic relatedness between the children was determined by verbal interviews with their mothers. A typical household (i.e., shared environment) consisted of a group of three to six adjacent houses. Within each household, the children formed several full-sibling, half-sibling, and first-cousin groups. The second study monitored severe malaria that led to hospitalization and nonmalaria hospitalizations in 2,900 children, also over a five-year period. This analysis concentrated on full-siblings.

Using a standard statistical genetics method of relating similarity in phenotype to similarity in genotype, they found that host genetic factors accounted for approximately one-quarter to one-third of the total variation in susceptibility in the populations to malaria. Of this percentage, only a small proportion could be attributed to the best known malaria resistance genes. This is consistent with other studies that suggest that malaria susceptibility is under the control of many different genes, with each individual gene having a relatively small epidemiological effect.

When assessing the contribution of household factors, the researchers found that for mild clinical malaria, those factors accounted for slightly more than a quarter of the total variation. For hospitalized malaria, they contributed about 15%, and for hospitalizations with fever that turned out not to be malaria, they contributed approximately 35%. Overall, children living in the 10% of households with the highest malaria incidence had approximately twice as many infections per year than those living in the 10% of households with the lowest incidence.

The researchers do not question the long-term benefits of understanding the genetic factors but conclude that “identifying and tackling the household effects must be the more efficient route to reducing the burden of disease in malaria-endemic areas.” Factors such as suitable conditions for mosquitoes to breed and survive as well as human behavior are likely to play major roles. “We need to determine what makes the difference between low-risk and high-risk households,” Mackinnon says, “but whatever it is, it seems likely to be an easy target using tools such as education and the low-cost, low-tech devices that we already have at hand such as bed nets, residual indoor spraying, and cleaning up backyards for mosquito breeding sites.”

